# Feasibility Evaluation of a Novel Bilateral Hand and Finger Training Device in Patients with Hand Dysfunction: A Preliminary Study

**DOI:** 10.3390/healthcare14182915

**Published:** 2026-09-09

**Authors:** Gaku Watanabe, Yukiyo Shimizu, Kei Takehara, Yuki Mataki, Shigeki Kubota, Yasushi Hada

**Affiliations:** 1 Doctoral Program in Medical Sciences, Graduate School of Comprehensive Human Sciences, University of Tsukuba, Tsukuba 305-8575, Ibaraki, Japan; watanabe.gaku.mv@ms.hosp.tsukuba.ac.jp (G.W.); takehara.kei.vr@ms.hosp.tsukuba.ac.jp (K.T.); 2 Department of Rehabilitation Medicine, University of Tsukuba Hospital, Tsukuba 305-8576, Ibaraki, Japan; 3 Department of Rehabilitation Medicine, Institute of Medicine, University of Tsukuba, Tsukuba 305-8575, Ibaraki, Japan; y-hada@md.tsukuba.ac.jp; 4 Department of Occupational Therapy, School of Health Sciences, Ibaraki Prefectural University of Health Sciences, Ami 300-0394, Ibaraki, Japan; kubotashi@ipu.ac.jp

**Keywords:** assistive technology, hand dysfunction, stroke, spinal cord injury, upper limb rehabilitation, rehabilitation device, bilateral training, feasibility study

## Abstract

**Background/Objectives**: The Synchronized Hand Interface for Neurorehabilitation and Innovation (SHINI) is a mechanical device that uses voluntary movement of the unaffected or less affected upper limb to generate synchronized bilateral hand and finger movements without biological signal detection or electronic control. This exploratory study evaluated SHINI’s technical operability and practical limitations during a single supervised session. **Methods**: Seven participants with hand dysfunction of varied etiologies attempted one 10 min session. Completion status was categorized as completed without therapist intervention, completed with therapist intervention, or not completed; no quantitative acceptability threshold was prespecified. Secondary outcomes were adverse events, overall perceived exertion and upper-limb fatigue assessed using the modified Borg Scale, exploratory pre- and post-session modified Ashworth Scale scores, and participant and therapist feedback. **Results**: Three participants (42.9%) completed the session without therapist intervention, three (42.9%) completed it with therapist intervention, and one (14.3%) did not complete it. Hand or forearm displacement affected four participants (57.1%), including the participant who discontinued. No adverse events occurred. Perceived exertion and upper-limb fatigue were generally low, no consistent direction of change in modified Ashworth Scale scores was observed, and feedback emphasized the need for improved fixation and adjustability. **Conclusions**: SHINI showed preliminary technical operability during a single supervised session, but the findings do not establish feasibility for independent or semi-supervised use. With improved fixation and adaptability, SHINI may offer a simple mechanical approach to repetitive bilateral hand training. Repeated-use studies are required to evaluate usability, safety, and clinical effectiveness.

## 1. Introduction

Hand dysfunction can result from stroke, spinal cord disorders, musculoskeletal and neuromuscular disorders, and trauma. It can restrict activities of daily living (ADLs), including eating, dressing, grooming, writing, and the use of electronic devices, thereby reducing independence and quality of life. Among these conditions, stroke is a major cause of persistent upper-limb and hand dysfunction. Approximately 12.2 million people experience a stroke worldwide each year [1]. Only 5–20% of patients with initial upper-limb impairment fully regain arm function, while 30–66% have no functional use of the affected upper limb six months after stroke [2]. Reduced upper-limb capacity is associated with dependence in ADLs and poor quality of life [3]. Rehabilitation is provided to improve upper-limb and hand function. Rehabilitation interventions include task-oriented training, repetitive practice, range-of-motion exercises, muscle strengthening, and interventions that promote use of the affected limb in daily life [4,5]. Evidence from stroke rehabilitation supports sufficient practice dose, repetition, task specificity, and active use of the affected limb as important principles for motor recovery [4,5]. Nevertheless, recovery of hand function often remains limited, particularly in patients with severe impairment [6]. Constraint-induced movement therapy is effective for selected patients but requires prolonged training and is generally limited to those with some residual voluntary movement [7,8]. Furthermore, the amount of upper-limb training provided in routine clinical practice is often limited and varies across settings [9,10]. Therapist time, staffing, equipment, and organizational priorities may restrict opportunities for repetitive practice [11,12]. In Japanese convalescent rehabilitation wards, improvement in ADLs is emphasized because ward performance is evaluated using an index based on motor Functional Independence Measure gain and length of stay [13]. Consequently, compensatory strategies using the non-paretic limb may be prioritized, particularly in patients with severe upper-limb impairment. Although compensation is important for independence, it may reduce opportunities to use the paretic upper limb and potentially limit its recovery [14].

Technology-assisted interventions, including robot-assisted rehabilitation and brain–computer interface (BCI)-based therapy, have been developed to increase opportunities for intensive and repetitive training. Meta-analyses have suggested beneficial effects on upper-limb motor function [15,16]. However, robot-assisted rehabilitation has not consistently demonstrated superiority over conventional rehabilitation [17]. Although BCI-based therapy has shown promising results, evidence regarding its long-term effects and the patients most likely to benefit remains limited [18,19]. In addition, widespread clinical implementation may be restricted by cost and the need for specialized equipment, biological signal detection and adjustment, and trained personnel [20].

Thus, a gap remains between conventional therapist-delivered rehabilitation and advanced technology-assisted interventions. There is a need for a device that can provide repetitive hand and finger movement with limited technical infrastructure and minimal therapist assistance. To address this need, we developed the Synchronized Hand Interface for Neurorehabilitation and Innovation (SHINI) in collaboration with THK Co., Ltd. SHINI uses voluntary movement of the unaffected or less affected upper limb to mechanically produce synchronized bilateral hand and finger movements without biological signal detection or electronic control. Although it was primarily developed for post-stroke hand dysfunction, its mechanically linked structure is not disease-specific, and the device may also be applicable to hand dysfunction caused by other conditions. Therefore, the present study was designed as an early evaluation of technical feasibility across varied causes and patterns of hand dysfunction. The aims were to evaluate the feasibility of a single supervised SHINI training session and to obtain feedback from participants and rehabilitation therapists to inform further refinement of the device.

## 2. Materials and Methods

### 2.1. Ethical Considerations

This study was conducted in accordance with the Declaration of Helsinki. Approval was obtained from the Ethics Committee of the University of Tsukuba Hospital before study commencement (approval no. R05-107). Written informed consent was obtained from all participants before enrollment. All participants also provided consent for publication, including the use of any accompanying images.

### 2.2. Participants

Participants were enrolled from among inpatients and outpatients receiving rehabilitation at the University of Tsukuba Hospital from November 2023 to January 2024. A convenience sampling approach was used. Potential participants were identified during routine rehabilitation, and patients who appeared to meet the eligibility criteria were invited to participate. Seven patients were invited; all seven met the eligibility criteria, provided written informed consent, and were enrolled. None declined participation. Because a formal screening log was not maintained, the total number of potentially eligible patients who were not approached could not be determined. The inclusion criteria were patients with hand dysfunction resulting from acquired brain disorders, spinal cord disorders, musculoskeletal disorders, neuromuscular disorders, or trauma.

The exclusion criteria were as follows: severe joint contractures or spasticity that made passive joint movement difficult; severe bilateral hand or upper-limb dysfunction that made use of the device difficult; and any condition judged by the investigators to make participation inappropriate.

Baseline clinical information, including age, sex, diagnosis, affected side, time since onset or injury, and available diagnosis-specific clinical assessments, was obtained from the medical records.

### 2.3. SHINI Intervention and Assessment Procedure

**Figure 1:**
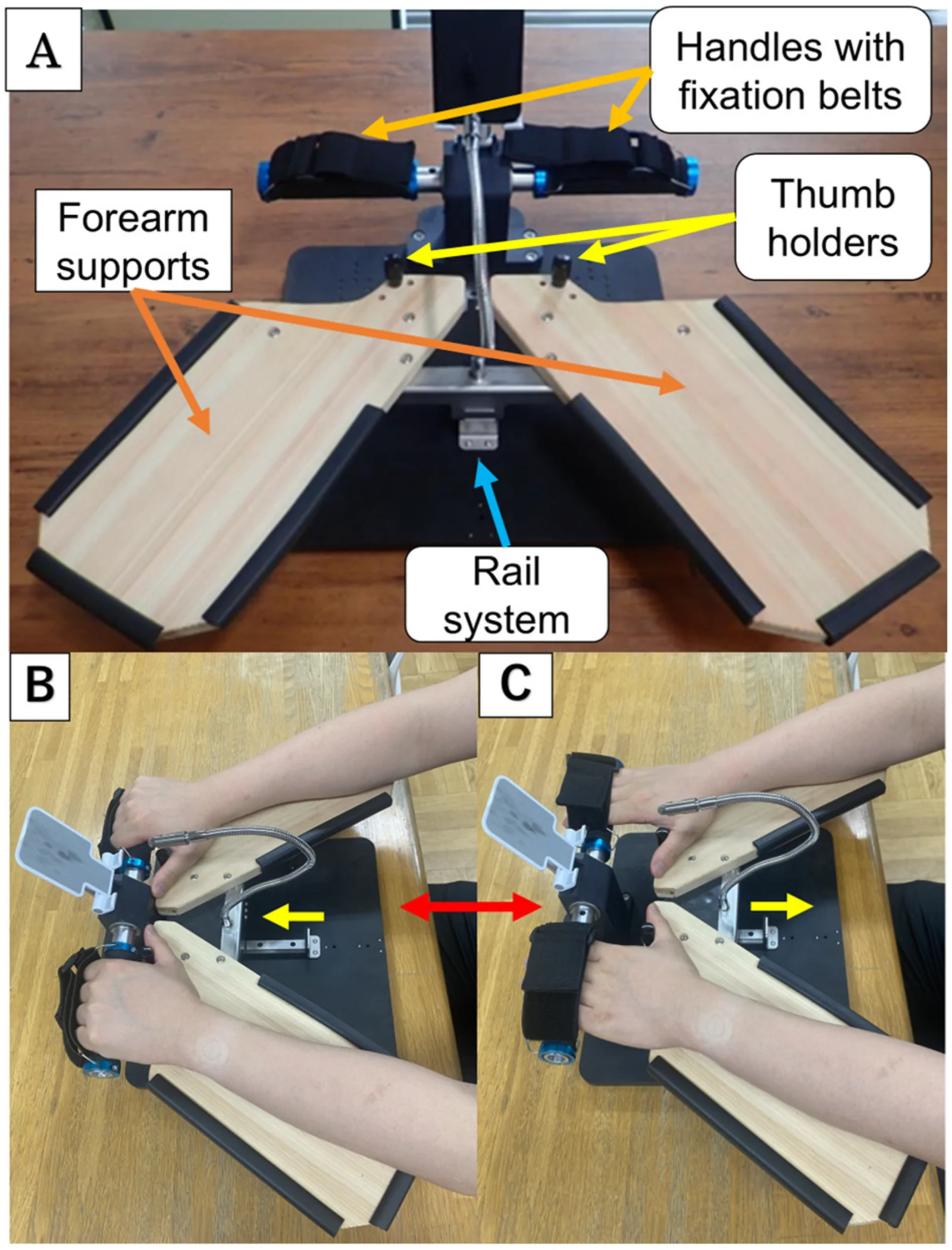
SHINI device and synchronized bilateral hand and finger movement during training. The participant’s hands are secured to the linked handles, and both forearms are positioned on the forearm supports. Panel (**A**) shows the overall structure of the device, and panels (**B**,**C**) show the two positions during forward and backward movement.

SHINI consists of bilateral handles with fixation belts, forearm supports mounted on a rail system, and thumb holders. SHINI is a prototype developed in collaboration with THK Co., Ltd. (Tokyo, Japan). The left and right forearm supports are mechanically connected. The left and right handles also rotate in a linked manner. During training, participants were instructed to move both upper limbs simultaneously. The unaffected or less affected forearm provided the driving force by moving forward and backward on the forearm support. Because these supports are mechanically linked, movement of the unaffected or less affected forearm simultaneously moved both supports together with both forearms and hands. With the fingers secured to the handles, this forward and backward movement caused the linked handles to rotate. This rotation converted the translational movement of the hands into synchronized flexion and extension of the fingers on both sides. The structure of the device is shown in Figure 1.

Before device setup, spasticity was assessed using the modified Ashworth Scale (MAS) [21], and overall perceived exertion and upper-limb fatigue were assessed using the modified Borg Scale (mBS) [22]. The device was then placed on a table in front of the participant, who was seated in a chair or wheelchair. The participant’s hands were secured to the handles with fixation belts, and the forearms were positioned on the forearm supports. After the therapists completed the device setup, participants trained with the device for 10 min at a self-selected pace. Setup time and positioning difficulty were not prospectively recorded. No participant-specific modifications were made to the device components.

Immediately after completion or discontinuation of the training session, the hands and forearms were released from the device, and the same assessments of spasticity, overall perceived exertion, and upper-limb fatigue were repeated. Participants and rehabilitation therapists completed separate questionnaires. Rehabilitation therapists also evaluated adverse events and feasibility.

### 2.4. Outcomes

The primary feasibility outcome was the completion status of the prescribed 10 min SHINI training session. The following three completion categories were prespecified in the research protocol: completed without therapist intervention, completed with therapist intervention, and not completed. Therapist intervention was defined as at least one additional hands-on action required after training had begun to allow the participant to continue the session, including repositioning the hand or forearm or re-securing the hand to the handle. A participant was classified as having completed the session with therapist intervention if any such action was required, regardless of its frequency or duration. The initial device setup performed before training was excluded from the definition of therapist intervention. The number and duration of therapist interventions were not prospectively recorded. “Not completed” indicated that the participant did not complete the prescribed 10 min training session. No quantitative or explicit operational criterion for discontinuation was prespecified. Although the three completion categories were prespecified, no quantitative threshold for acceptable feasibility was established before the study.

**Figure 2:**
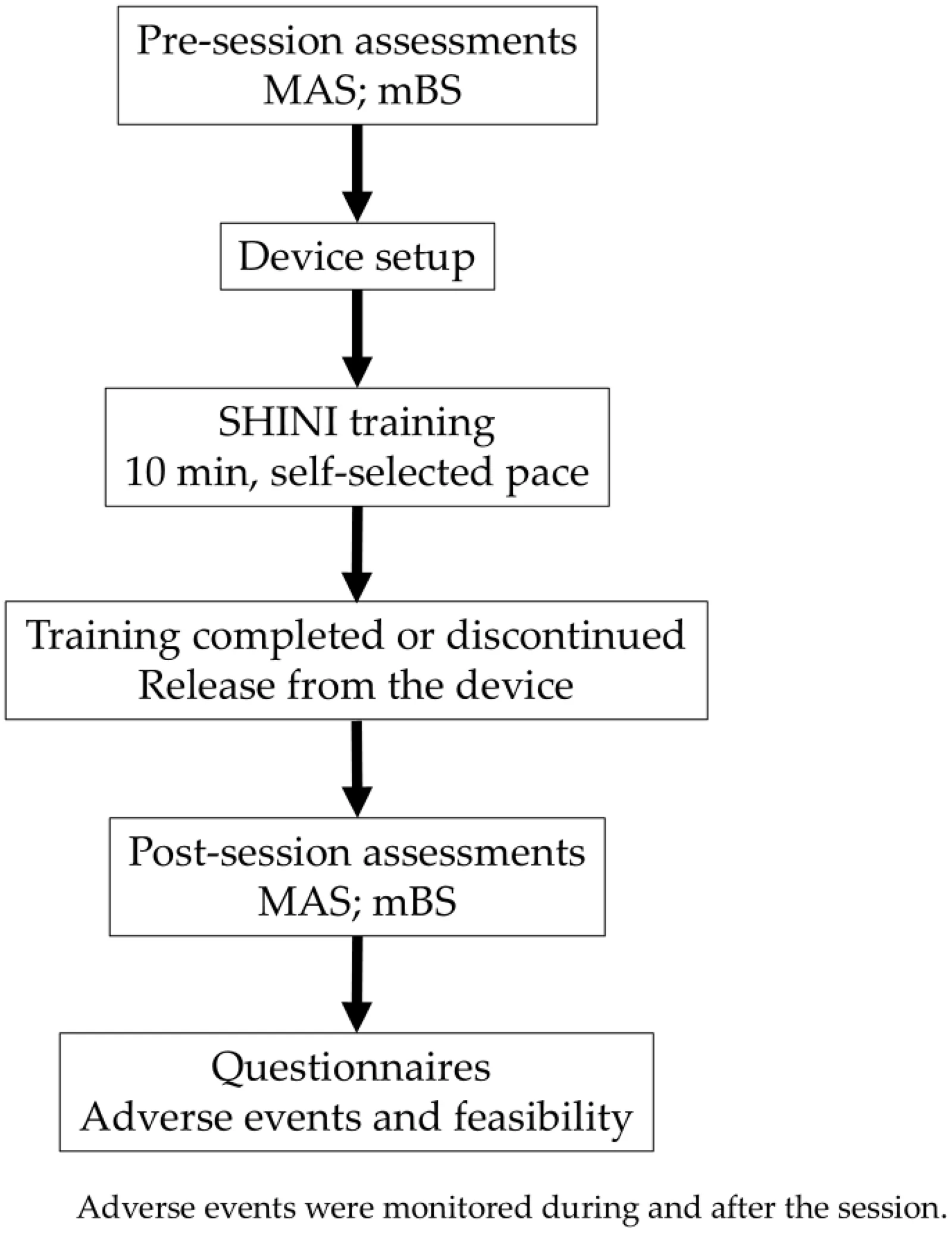
Study flow and measurement time points. MAS, Modified Ashworth Scale; mBS, modified Borg Scale.

Secondary outcomes were adverse events, modified Borg Scale (mBS) scores for overall perceived exertion and upper-limb fatigue, spasticity, and questionnaire responses. Spasticity was assessed using the modified Ashworth Scale (MAS) for shoulder flexion, shoulder extension, shoulder abduction, elbow flexion, elbow extension, wrist flexion, wrist extension, forearm pronation, forearm supination, finger flexion, and finger extension. Pre- and post-session MAS assessments were prespecified in the research protocol to document muscle tone and explore the direction of any immediate changes after a single training session, thereby providing preliminary information for the design of future studies in patients with stroke. The same assessment protocol was applied to all participants. These assessments were exploratory and were not intended to determine therapeutic efficacy. Because the MAS has limited clinical relevance in participants with non-neurological causes of hand dysfunction, the MAS scores were interpreted descriptively. Separate questionnaires were administered to participants and therapists. The participant questionnaire included open-ended questions regarding training duration, ease of use, device design, and potential improvements to the forearm supports, hand fixation, handles, and thumb holders. Participants were also asked whether they would like to continue using the device, with a yes-or-no response option, and were provided with an additional section for unrestricted comments. The therapist questionnaire included open-ended questions regarding ease of preparation and setup, device design, and potential improvements to the forearm supports, hand fixation, handles, and thumb holders, together with an additional section for unrestricted comments. These study-specific questionnaires were developed for this preliminary feasibility study and were not formally validated. They were used to collect exploratory feedback from participants and therapists. The measurement time points and study flow are shown in Figure 2.

### 2.5. Statistical Analysis

Because this was an exploratory feasibility study and was not designed to estimate a treatment effect, no formal sample size calculation was performed. All outcomes were summarized descriptively. The available clinical assessments differed among diagnoses and were therefore reported descriptively. Continuous variables were presented as means with standard deviations, medians with ranges, or individual values, as appropriate. Categorical variables were presented as counts and percentages. The three feasibility completion categories were summarized using counts and percentages. MAS scores were reported descriptively at the individual-participant level. No inferential statistical testing was performed.

Because the study had no comparison group and was not designed to evaluate clinical effectiveness, intervention effect estimates and confidence intervals were not calculated.

One investigator reviewed the open-ended questionnaire responses after data collection. Responses were organized according to the questionnaire items and summarized descriptively based on similarities in content. No formal qualitative analysis or coding procedure was used. Descriptive statistics were calculated using Microsoft Excel for Microsoft 365 (Microsoft Corporation, Redmond, WA, USA).

## 3. Results

### 3.1. Participant Characteristics

Seven patients were invited to participate. All seven met the eligibility criteria, provided written informed consent, and were enrolled; none declined participation. All seven received the intervention and were included in the descriptive analysis. The number of patients considered as potential candidates before invitation was not recorded, as no formal screening log was maintained. Participant characteristics and available baseline clinical findings are shown in Table 1. The mean age was 54.9 ± 18.1 years (range, 26–75). The median age was 56 years. The participants comprised four men (57.1%) and three women (42.9%). The median time since onset or injury was 1.6 years (range, 0.1–55.0 years). The underlying conditions included acquired brain disorders in three participants (42.9%): sequelae of encephalitis, cerebral infarction, and putaminal hemorrhage. Two participants (28.6%) had spinal cord disorders, including central cord syndrome and cervical spondylotic myelopathy. The remaining two participants (28.6%) had trigger fingers and thermal crush injury of the right hand, respectively. Both participants with spinal cord disorders were classified as neurological level of injury C5 and American Spinal Injury Association Impairment Scale grade D. Among these two participants, one had unilateral upper-limb dysfunction on the left side, whereas the other had bilateral upper-limb dysfunction with left-sided predominance. The 55-year time since onset reported for Participant 1 reflected encephalitis that occurred in her childhood. At the time of the study, this participant had long-standing right hemiparesis and spasticity and was receiving orthotic management and botulinum toxin treatment.

### 3.2. Feasibility and Safety

**Figure 3:**
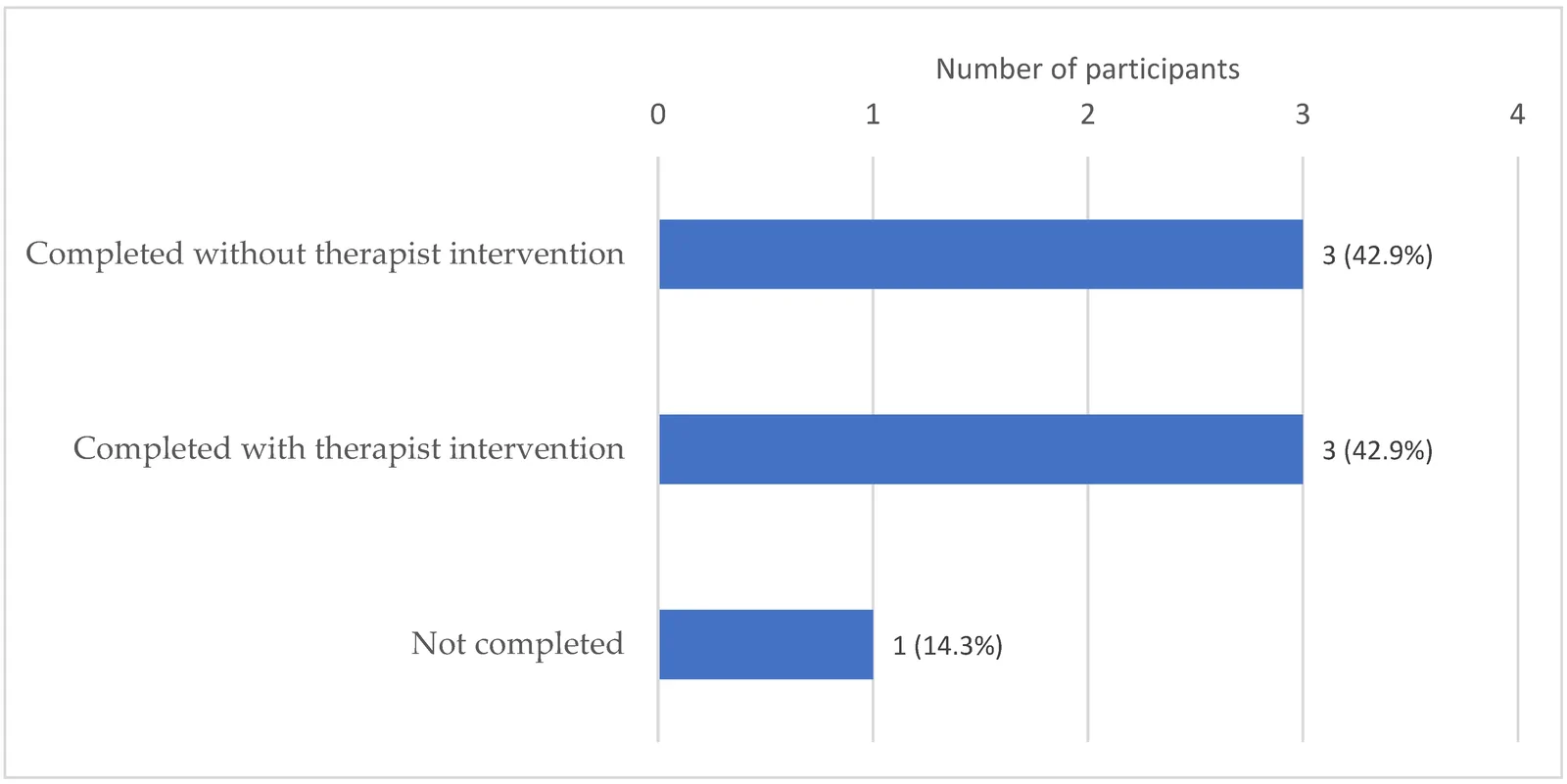
Feasibility outcome: training completion status. Values are presented as *n* (%). Percentages do not sum to 100% because of rounding.

Feasibility, safety outcomes, and modified Borg Scale scores are shown in Table 2. Six of the seven participants completed the prescribed 10 min training session. Three participants (42.9%) completed the session without therapist intervention, whereas three (42.9%) completed it with therapist intervention. One participant (14.3%) did not complete the session. Thus, three of the six participants who completed the session required therapist intervention. The distribution of completion status is summarized in Figure 3.

The three participants who required therapist intervention needed repositioning of the forearm and hand and re-securing of the hand to the handle because displacement occurred during training. Including the participant who did not complete the session, hand or forearm displacement affected four of seven participants (57.1%). The participant who did not complete the session had severe hemiparesis. Marked displacement of the paretic forearm and hand occurred during training, making it difficult to maintain stable use of the device despite repeated repositioning and other adjustments by the therapist. The therapist judged that the session could not be continued as intended, and the investigator agreed; the session was therefore discontinued.

No adverse events were observed in any participant during or after training (0/7, 0%).

### 3.3. Modified Borg Scale and Modified Ashworth Scale

The median mBS score for overall perceived exertion was 0 (range, 0–0) before training and 0 (range, 0–2) after training. The median mBS score for upper-limb fatigue was 0 (range, 0–0) before training and 0.5 (range, 0–2) after training. Individual mBS scores are shown in Table 2.

Individual pre- and post-session MAS scores are presented in Supplementary Table S1. No consistent direction of change in MAS scores was observed after the training session.

### 3.4. Questionnaire Responses

Questionnaire responses from participants and rehabilitation therapists are presented separately in Table 3. All seven participants (100%) indicated that they would like to continue using SHINI. Five participants (71.4%) reported that the device was easy to use, one (14.3%) stated that it would be easier to use if hand fixation were improved, and one (14.3%) did not respond. Four participants (57.1%) considered the 10 min training duration appropriate, one (14.3%) considered it too long, one (14.3%) considered it too short, and one (14.3%) did not respond. Participant comments mainly concerned hand fixation, forearm-support position, and the size or position of the handles and thumb holders.

Therapist comments focused on clinical setup and adaptation to different patients. They identified device weight and portability, insufficient fixation of the paretic hand or wrist, and the need to accommodate contracture, spasticity, or hand deformity. They also suggested adjustable forearm supports, handles, and thumb holders, as well as resistance, assistance, joint-specific, and game-based training modes.

## 4. Discussion

In this feasibility study, six of seven participants completed a single 10 min session of SHINI training; however, only three completed the session without therapist intervention, whereas three required therapist intervention. One participant did not complete the session because stable use of the device could not be maintained. No adverse events were observed during or after the session. Overall perceived exertion and upper-limb fatigue assessed using the modified Borg Scale were generally low, and no consistent direction of change in modified Ashworth Scale scores was observed after training. In addition, feedback from participants and rehabilitation therapists identified several areas for device refinement. These findings provide preliminary evidence of SHINI’s technical operability during a single supervised session in this small sample but do not establish that the device can currently be used independently or with limited supervision.

Hand or forearm displacement affected four of seven participants and was the principal barrier to stable use of the device. Furthermore, because no quantitative threshold for acceptable feasibility was established before the study, the results are descriptive and do not demonstrate that a predefined feasibility criterion was met. The need for therapist intervention is clinically important because limited therapist assistance is one of the intended advantages of SHINI. Three of the six participants who completed training required at least one hands-on intervention for repositioning or re-securing the hand or forearm. Although any hands-on intervention was counted, its frequency and duration were not prospectively recorded. Therefore, the actual burden placed on the therapist could not be quantified. Setup time and positioning difficulty were also not prospectively recorded. Consequently, the present study did not directly evaluate whether SHINI can be set up quickly or used with minimal therapist involvement. Future studies should prospectively record setup time and the number, duration, and reasons for therapist interventions.

The participant who did not complete training had severe hemiparesis, with BRS II in the upper limb and BRS I in the fingers. Marked displacement of the paretic hand and forearm prevented stable use of the device despite therapist repositioning and re-securing. This finding suggests that the current fixation system may have limited applicability to patients with very limited voluntary hand control. However, patient-selection criteria cannot be established from a single participant. Future studies should examine device use according to baseline voluntary motor control, muscle strength, spasticity, contracture, sensory impairment, and hand deformity. Stronger or more adaptable fixation and additional forearm and wrist support may be required for patients with severe impairment.

A key feature of SHINI is that it generates synchronized bilateral hand and finger movements through a mechanically linked system. The user attempts to move both upper limbs simultaneously, while the unaffected or less affected limb provides the force that moves the affected hand. This mechanism is conceptually related to bilateral arm training, in which synchronized bilateral movement may recruit motor networks in both hemispheres and modulate interhemispheric inhibition [23,24]. However, cortical activity and interhemispheric inhibition were not assessed in the present study.

The temporal association between motor intention and assisted movement is also relevant to the design of SHINI. Unlike mirror therapy, which provides the visual impression of affected-limb movement [25], SHINI produces actual movement of the affected hand and accompanying somatosensory feedback. The Hybrid Assistive Limb, a wearable robotic device, and BCI-based interventions use biological signals related to voluntary movement or motor intention to trigger assistance [26,27,28], whereas SHINI achieves temporal coupling through a mechanical linkage. This coupling may link motor intention with somatosensory feedback and thereby support the sensorimotor loop and activity-dependent neuroplasticity [29,30]. However, these mechanisms were not directly assessed and should be regarded as hypotheses for future research rather than findings of the present study.

Although SHINI was originally developed for post-stroke hand dysfunction, this early feasibility study included participants with varied conditions to examine its technical use in the presence of different patterns of weakness, spasticity, contracture, pain, ataxia, and sensory impairment. This approach provided information relevant to prototype refinement, but the clinical heterogeneity limits diagnosis-specific interpretation. One participant had a 55-year history of hemiparesis following childhood encephalitis. Although the long-standing unilateral hemiparesis and spasticity were clinically comparable in some respects to chronic stroke, the different etiology and exceptionally long time since onset should be considered when comparing this participant with the others. Future studies should evaluate larger and more clinically homogeneous populations, including patients with stroke at defined stages of recovery.

The pre- and post-session MAS assessments were exploratory and were included to document muscle tone and examine the direction of any immediate changes, thereby providing preliminary information for the design of future studies in patients with stroke. No consistent direction of change was observed after the single 10 min session. Given the short exposure and the limited clinical relevance of the MAS in participants with non-neurological hand dysfunction, these findings should not be interpreted as evidence of an immediate therapeutic effect on spasticity. Nevertheless, these observations may help inform the selection of outcome measures and assessment procedures in future studies involving patients with stroke.

The questionnaire responses provided important information from two different perspectives. All seven participants indicated that they would like to continue using SHINI, and most reported that it was easy to use. These responses may reflect favorable initial acceptability after a single training session. However, they were obtained immediately after one supervised session using study-specific questionnaires that had not been formally validated. Responses may have also been influenced by the involvement of the investigators in device development. Therefore, sustained acceptability and willingness to continue using the device should be interpreted with caution and evaluated during repeated use. Participant comments mainly concerned hand fixation and the fit and position of the forearm supports, handles, and thumb holders. In contrast, therapist comments focused on clinical setup, portability, fixation of the paretic hand or wrist, and adaptation to contracture, spasticity, or hand deformity. Therapists also suggested adjustable components and additional training modes. These comments were consistent with the observed displacement and fixation problems during training. Thus, improved fixation, adjustability, and adaptability should be considered priorities for further prototype development. Independent or semi-supervised use should be evaluated only after these issues have been addressed and stable operation has been demonstrated during repeated sessions.

This study has several limitations. First, the sample was small, clinically heterogeneous, and recruited using convenience sampling. Because no formal screening log was maintained, the number and characteristics of potentially eligible patients who were not approached could not be determined, and selection bias cannot be excluded. Second, the study was conducted at a single center by investigators involved in developing the device. The assessments were not blinded or performed by independent assessors, which may have introduced investigator bias. Future studies should include evaluation by independent investigators. Third, baseline clinical assessments differed according to diagnosis, and standardized measures of upper-limb and hand impairment were not available for all participants. Fourth, no quantitative threshold for acceptable feasibility or explicit operational criterion for discontinuation was prespecified. Fifth, setup time and the frequency and duration of therapist interventions were not prospectively recorded. Sixth, SHINI was evaluated during only one short supervised session; therefore, feasibility, safety, adherence, and user experience during repeated or long-term use remain unknown. Seventh, the study did not evaluate clinical effectiveness using standardized functional outcomes or directly assess the proposed neurophysiological mechanisms or sense of agency. Finally, the MAS was applied to participants with both neurological and non-neurological conditions, and the study-specific questionnaires were not formally validated.

Taken together, the present findings indicate preliminary operability of SHINI during a single supervised session, while fixation and adaptability emerged as the main barriers to stable and independent use. Further device refinement and studies involving prospectively recorded therapist involvement, repeated training sessions, and larger, clinically more homogeneous samples are required.

## 5. Conclusions

Six of seven participants completed a single supervised 10 min SHINI training session; however, only three completed it without therapist intervention. Hand or forearm displacement affected four participants, including one who could not complete the session, while no adverse events were observed. These findings provide preliminary evidence of SHINI’s technical operability during a single supervised session in this small, clinically heterogeneous sample but also identify fixation and adaptability as the main barriers to stable and independent use. Further device refinement and repeated-use studies are required to evaluate usability, safety, and the potential for independent or semi-supervised use. Clinical effectiveness should be examined in subsequent studies.

## 6. Patents

A patent application related to the device evaluated in this study has been filed by THK Co., Ltd. and laid open as Japanese Unexamined Patent Application Publication No. JP 2024-70823 A (published 23 May 2024); the addition of G.W. and Y.S. as inventors is currently in progress. A separate patent application for a successor device has been filed, on which G.W., Y.S., and Y.H. are inventors.

## Figures and Tables

**Table 1:** Participant characteristics and available baseline clinical findings.

Participant	Age (Years)	Sex	Condition	Affected Side	Time Since Onset or Injury (Years)	Baseline Clinical Findings
1	56	Woman	Sequelae of childhood encephalitis	Right	55.0	Right hemiparesis BRS: UL III, F II, LL III
2	26	Woman	Thermal crush injury of the right hand	Right	1.2	Mild finger joint contractures and hand weakness (grip strength: 12.5 kg)
3	74	Man	Central cord syndrome (postoperative) NLI: C5; AIS: D	Left	0.1	Left-predominant mild upper-limb weakness (MMT grade approximately 4; grip strength: 5.0 kg)
4	64	Man	Trigger finger of the index, middle, and ring fingers (postoperative)	Left	1.6	Mild finger joint contracture in the index, middle, and ring fingers
5	50	Woman	Cerebral infarction	Left	8.0	Left hemiparesis BRS: UL IV, F III, LL V
6	39	Man	Right putaminal hemorrhage	Left	0.1	Left hemiparesis BRS: UL II, F I, LL III
7	75	Man	Cervical spondylotic myelopathy (postoperative) NLI: C5; AIS: D	Bilateral (left-predominant)	3.2	Bilateral upper-limb weakness Finger extension (MMT): grade 4 (right), grade 2 (left) Reduced sensation distal to both forearms

BRS, Brunnstrom Recovery Stage; UL, upper limb; F, finger; LL, lower limb; MMT, manual muscle testing; NLI, neurological level of injury; AIS, American Spinal Injury Association Impairment Scale. Time since onset or injury was calculated from the onset of the condition or from the injury to the study session, not from the date of any preceding surgery.

**Table 2:** Results of feasibility, safety, and modified Borg Scale assessments.

Participant	Feasibility	Reason for Therapist Intervention or Non-Completion	Adverse Events	Overall Exertion, mBS (Pre/Post)	Upper-Limb Fatigue, mBS (Pre/Post)
1	Completed with therapist intervention	Repositioning and re-securing due to displacement	None	0/0	0/1
2	Completed without therapist intervention	N/A	None	0/0	0/0
3	Completed without therapist intervention	N/A	None	0/2	0/2
4	Completed without therapist intervention	N/A	None	0/0	0/0.5
5	Completed with therapist intervention	Repositioning and re-securing due to displacement	None	0/0	0/0
6	Not completed	Stable use could not be maintained despite repeated adjustment	None	0/0	0/0
7	Completed with therapist intervention	Repositioning and re-securing due to displacement	None	0/0	0/2

mBS scores are presented in the order pre-session/post-session. mBS, modified Borg Scale; N/A, not applicable.

**Table 3:** Questionnaire responses from participants and rehabilitation therapists.

Question/Domain	Summary of Responses
**Participant responses**	
Training duration	Appropriate: 4; too long: 1; too short: 1; no response: 1
Ease of use	Easy to use: 5; easier if hand fixation were improved: 1; no response: 1
Willingness to continue	Yes: 7
Forearm supports	Easier arm placement; adjustable height and angle; wider movement range
Hand fixation	More secure fixation; prevention of loosening or displacement during training
Handles and thumb holders	Adjustable handle size; softer handle surface; adjustable or additional thumb holder
Other comments	Improved table stability; less device noise; adjustable movement or resistance
**Therapist responses**	
Portability	Reduced device weight; carrying handles or an easier way to carry the device
Setup and stability	Generally simple and stable; anti-slip measures needed in some cases
Forearm supports	Additional fixation when needed; adjustable height; wider movement range
Hand and wrist fixation	Stronger fixation for paretic hands; adaptation for contracture or spasticity; wrist fixation
Handles and thumb holders	Adjustable size, shape, and position; alternatives for different hand deformities
Training functions and design	Resistance and assistance modes; joint-specific modes; application or game-based training; improved display design

## Data Availability

The data presented in this study are available on request from the corresponding author. The data are not publicly available because the small sample size and the individual-level clinical data reported could allow participants to be identified.

## Supplementary Materials

The following supporting information can be downloaded at: https://www.mdpi.com/article/10.3390/healthcare14182915/s1, Table S1: Individual pre- and post-session modified Ashworth Scale scores.
