# Supplementary material for: Feasibility Evaluation of a Novel Bilateral Hand and Finger Training Device in Patients with Hand Dysfunction: A Preliminary Study
Source: Healthcare (Basel). 2026 Sep 9;14(18):2915. doi: 10.3390/healthcare14182915 (PMC13606373; doi:10.3390/healthcare14182915)
Supplement: Supplementary file 1 [file healthcare-14-02915-s001.zip › healthcare-4505546-supplementary.pdf]

Table S1. Individual pre- and post-session modified Ashworth Scale (MAS) scores.

A. Proximal upper limb

Participant	Assessed side	Shoulder flexion	Shoulder extension	Shoulder abduction	Elbow flexion	Elbow extension
1	Right	1+ → 1+	0 → 0	1+ → 1+	1 → 0	1+ → 2
2	Right	0 → 0	0 → 0	0 → 0	0 → 0	0 → 0
3	Left	0 → 0	0 → 0	0 → 0	0 → 0	1 → 0
4	Left	0 → 0	0 → 0	0 → 0	0 → 0	0 → 0
5	Left	1 → 1	0 → 0	1 → 1	0 → 0	2 → 2
6	Left	0 → 0	0 → 0	0 → 0	0 → 0	1+ → 1+
7	Right	0 → 0	0 → 0	0 → 0	0 → 0	0 → 0
7	Left	0 → 0	0 → 0	0 → 0	0 → 0	0 → 0

B. Distal upper limb

Participant	Assessed side	Wrist flexion	Wrist extension	Forearm pronation	Forearm supination	Finger flexion	Finger extension
1	Right	0 → 0	0 → 1	0 → 0	1+ → 1+	2 → 1+	1 → 1
2	Right	0 → 0	0 → 0	0 → 0	0 → 0	0 → 0	0 → 0
3	Left	0 → 0	0 → 0	0 → 0	0 → 0	0 → 0	0 → 0
4	Left	0 → 0	0 → 0	0 → 0	0 → 0	0 → 0	0 → 0
5	Left	1 → 1	1+ → 1+	0 → 0	1+ → 1+	1 → 1	1+ → 1+
6	Left	0 → 0	1+ → 1	0 → 0	1 → 0	0 → 0	2 → 2
7	Right	0 → 0	0 → 0	0 → 0	0 → 0	0 → 0	0 → 0
7	Left	0 → 0	0 → 0	0 → 0	0 → 0	0 → 0	0 → 0

Values are presented in the order pre-session → post-session. For participants with unilateral impairment, only the affected side is shown; for Participant 7, who had bilateral upper-limb impairment, both sides are shown. For Participant 6, who did not complete the session, the post-session assessment was performed immediately after discontinuation.
